# Whole-Body Cryostimulation: An Effective Complementary Treatment in Fibromyalgia? A Follow Up Study

**DOI:** 10.3390/jpm14080836

**Published:** 2024-08-07

**Authors:** Federica Verme, Neža Majdič, Giuseppe Modaffari, Angelo Alito, Alessandra Scarpa, Paolo Piterà, Amelia Brunani, Jacopo Maria Fontana, Paolo Capodaglio

**Affiliations:** 1 Research Laboratory in Biomechanics, Rehabilitation and Ergonomics, IRCCS, Istituto Auxologico Italiano, 28824 Verbania, Italy; f.verme@auxologico.it (F.V.); docmodaffarigiuse@gmail.com (G.M.); brunani@auxologico.it (A.B.); p.capodaglio@auxologico.it (P.C.); 2 Orthopedic Hospital Valdoltra, 6280 Ankaran, Slovenia; neza.majdic@ob-valdoltra.si; 3 Outpatient Rehabilitation Service, University Rehabilitation Institution Republic of Slovenia Soča, 1000 Ljubljana, Slovenia; 4 Department of Biomedical, Dental Sciences and Morphological and Functional Images, University of Messina, 98125 Messina, Italy; alitoa@unime.it; 5 Psychology Research Laboratory, IRCCS, Istituto Auxologico Italiano, 20145 Milano, Italy; 6 Department of Clinical and Biological Sciences, University of Turin, Orbassano, 10043 Torino, Italy; p.pitera@auxologico.it; 7 Department of Surgical Sciences, Physical and Rehabilitation Medicine, University of Torino, 10126 Torino, Italy

**Keywords:** chronic pain, disease activity, fibromyalgia, follow-up, long-term effects, rehabilitation, whole-body cryostimulation

## Abstract

Recent evidence suggests that whole-body cryostimulation (WBC) may be beneficial for patients with fibromyalgia (FM), but little is known about the duration of such effects. The purpose of this study was to verify the duration of clinical–functional benefits after one cycle of WBC. We conducted a follow-up study on the medium and long-term effects of WBC on well-being, use of pain-relieving/anti-inflammatory medications, pain level, fatigue, sleep quality, and psychological aspects such as mood and anxiety. Twelve months after discharge, we administered a 10 min follow-up telephone interview with FM patients with obesity who had undergone ten 2 min WBC sessions at −110 °C as part of a multidisciplinary rehabilitation program (*n* = 23) and with patients who had undergone rehabilitation alone (*n* = 23). Both groups reported positive changes after the rehabilitation program, and similar results regarding fatigue, mood, and anxiety scores; however, the implementation of ten sessions of WBC over two weeks produced additional benefits in pain, general well-being status, and sleep quality with beneficial effects lasting 3–4 months. Therefore, our findings suggest that adding WBC to a rehabilitation program could exert stronger positive effects to improve key aspects of FM such as general well-being, pain level, and sleep quality.

## 1. Introduction

Fibromyalgia (FM) is a medical debilitating condition of unknown aetiology mainly characterised by chronic widespread musculoskeletal pain [1]. FM classification criteria based on the 2016 revisions of the American College of Rheumatology (ACR) diagnostic criteria 2010/2011 [2] and the diagnostic criteria of the ACTTION-APS pain taxonomy [3] include severe fatigue, morning stiffness, sleep disturbances (e.g., insomnia, frequent awakenings, and nonrestorative sleep), autonomic disturbances, hypersensitivity to external stimuli and memory deficits may also be present, reducing people’s quality of life and limiting social, occupational, and recreational activities [4,5,6]. In addition, patients with FM often report depressive symptomatology [6,7], which could be ascribed to chronic pain and related limitations [8,9]. 

FM is more common in women, with prevalence ranging from 3.3% to 8.3% in Europe [1], requiring a multidisciplinary therapeutic approach including pharmacological treatments and non-pharmacological measures [4,10]. Before prescribing medication, the first step in managing patients with FM is educating them about the condition, its symptoms, and available therapies. They should also be encouraged to exercise frequently, eat a balanced diet, and come up with their own methods and strategies for enhancing their quality of life [4]. 

The complex and poorly understood aetiology of FM, together with the wide range of signs and symptoms and multiple comorbidities, make the identification and design of effective therapies particularly challenging [11]. As a result, there is no gold standard on the best therapeutic approach or medications, making the treatment of FM a challenge for physicians. A combination of drugs, usually antidepressants, anticonvulsants, anti-inflammatories, and antioxidants, can be used [4,12,13,14], but non-pharmacological treatment measures can also be taken to manage this condition which include physiotherapy, aerobic and anaerobic training, and psychotherapy, including cognitive-behavioural interventions, biofeedback, and psychological support [4,15,16]. In addition, some studies have demonstrated beneficial effects of nutritional interventions on FM symptoms [17], but given the still limited evidence on the topic, further research is needed [18,19]. However, at present, neither pharmacological nor nonpharmacological treatments alone provide optimal results. 

Management of FM symptoms can be costly in the long run necessitating new complementary strategies that can prolong beneficial effects. Integrated approaches, aiming at reducing pain levels and improving overall functioning, which include both nonpharmacological and pharmacological therapies, have been shown to improve outcomes in FM patients [20,21]. The typical components of these multidisciplinary programmes for FM usually include educational, cognitive and behavioural interventions, physical training [22], and medication [23]. 

Taking into account the prevalence and significant economic impact of rheumatic diseases such as FM—whose costs are even higher than those of other chronic diseases such as cardiovascular disease and cancer [24]—it is important to develop and implement multidisciplinary treatments for FM that are methodologically rigorous and outweigh the limitations of previous studies. 

Emerging evidence has shown that whole-body cryostimulation (WBC) could represent a promising adjuvant treatment to address the dots in this vicious cycle between pain, mood, and sleep in various conditions of rehabilitation interest. 

WBC is a physical treatment which consists of exposing the entire body to cryogenic temperatures (−110 °C to −140 °C) for 2–3 min. Exposure to these temperatures is able to lower pain and inflammation, improving several metabolic parameters (thermogenesis, lipid profile, insulin sensitivity, and glucose utilisation) [25,26,27], as well as depression, anxiety [28], and sleep quality [29]. Furthermore, cycles of WBC have been shown to reduce fatigue and disease activity in patients with several conditions, such as multiple sclerosis [30], post-COVID-19 condition (PCC) [31], rheumatoid arthritis [32,33], polymyalgia rheumatica (PMR) [34], and fibromyalgia [9,35,36].

The majority of research to date has focused on how WBC affects FM, decreasing pain intensity [37,38], disease impact [37], and improving quality of life [37,38,39]. Indeed, preliminary evidence suggests that WBC may also positively affect sleep quality [39,40] and physical functioning [41], and may reduce depressive symptoms [28]. Patients with FM and comorbid obesity, who have higher levels of pain severity, depressive symptoms, impact of disease, and poorer sleep quality, can particularly benefit from WBC treatments. This hypothesis was confirmed in a recent study by our group [9], where the implementation of 10 WBC sessions within a multidisciplinary rehabilitation program resulted in greater improvements in patients’ severity of pain, depressive symptoms, disease impact, and sleep quality compared to the control group (who underwent rehabilitation only), thus suggesting that WBC could act as a promising add-on treatment to improve the main FM symptoms. However, most of the literature on the clinical use of WBC in patients with FM does not investigate the duration of its effects. In fact, only two studies have performed follow-up investigations [35,39], showing for the first time that WBC can have a lasting effect.

To verify the duration of clinical–functional benefits after a cycle of 10 WBC treatments, we conducted a follow-up study on the medium and long-term effects of WBC on well-being, use of pain-relieving/anti-inflammatory medications, pain level, fatigue, sleep quality, and psychological aspects (mood and anxiety). Twelve months after discharge, we conducted 10 min follow-up telephone interviews with FM patients who had undergone 10 WBC treatments as part of a multidisciplinary rehabilitation program and with patients who had undergone rehabilitation alone. Subjects from the cohort of interviewees who had participated in one of our previous studies on FM [9] were also included, and new subjects were added.

## 2. Materials and Methods

**Figure 1:**
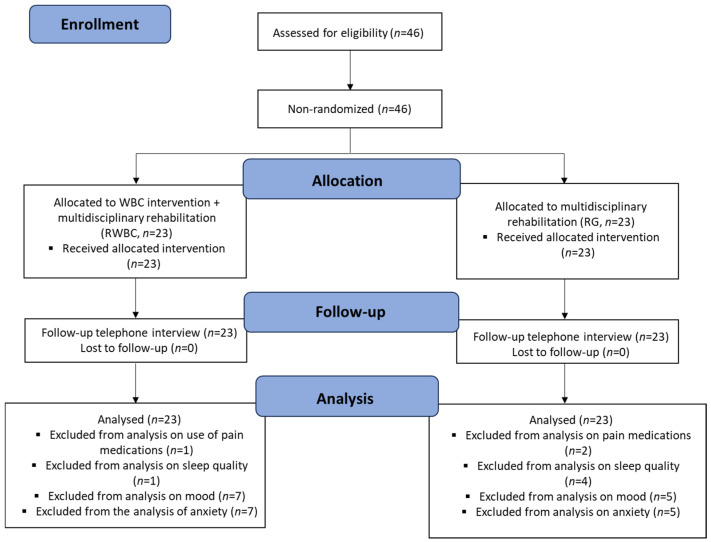
CONSORT flow chart describes the different stages of the study: patients’ enrollment, allocation into two different groups, the follow-up stage, and data analysis.

All eligible patients were consecutively recruited from the in-patients of the Rehabilitation Unit of the IRCCS Istituto Auxologico Italiano (Piancavallo, Verbania, Italy). Within a cohort of 46 women with FM and obesity (see Table 1) undergoing a 4-week multidisciplinary rehabilitation program, participants were consecutively, non-randomly, assigned to either a “rehabilitation + WBC group” (RWBC, *n* = 23), or a “rehabilitation-only group” (RG, *n* = 23). The stages of the protocol are described in Figure 1 and include patients’ enrollment, allocation, follow-up, and data analyses. Data were collected at three time points, at the time of hospital admission before the start of the rehabilitation program (T1), four weeks later upon discharge (T2) and 12 months after discharge. Some patients were unable to answer some of the questions asked during the interview, mainly due to memory bias. Unanswered questions were therefore excluded from the statistical analysis.

Both groups carried out a rehabilitation program aimed at weight management and physical conditioning, which included nutritional intervention, psychological support, physiotherapy, and physical activity. In addition to rehabilitation, the RWBC group underwent 10 sessions of WBC (once daily at −110 °C/2 min) in a cryo-chamber (Artic, CryoScience, Rome, Italy). Before starting the treatments, patients were examined by a medical doctor to rule out any contraindications according to Bad Voslau’s guidelines [42]. Moreover, they were instructed to remove glasses and metal accessories and to wear a T-shirt, running shorts, an earmuff band, gloves, socks (pulled up to the knee) and rubber slippers. 

The daily program consisted of physical therapy sessions, nutritional and psychological support, adapted physical activity classes and was given by a healthcare provider. The following inclusion criteria were applied: (i) age between 18 and 80 years; (ii) FM diagnosed by a rheumatologist according to the criteria of the American College of Rheumatology; (iii) FM diagnosed for more than one year; and meet the criteria for finding FM as measured by the Fibromyalgia Survey Questionnaire, Italian version 21. The exclusion criteria were as follows: (i) severe psychiatric conditions, (ii) acute respiratory disease, acute cardiovascular disease, unstable hypertension, cold intolerance, claustrophobia, pregnancy, (iii) recent modification of usual drug treatment, (iv) previous WBC, and (v) body temperature above 37.5 °C. This study was an open trial, so both participants and researchers were aware of what treatment (i.e., WBC) the patient was receiving. Written and verbal communications about the research protocol were given to each participant who, if agreeing to take part in the study, signed an informed consent form explaining the study procedures, in accordance with the Declaration of Helsinki. The Ethics Committee of the Istituto Auxologico Italiano approved the study protocol and materials (code 2021_05_18_14). The sample analysed in this study represents a subset of a larger cohort originally collected to investigate broader trends in the impact of WBC in patients with metabolic or neurological disease or FM. The subset chosen for this specific analysis was selected based on a diagnosis of FM, with either WBC treatments and multidisciplinary rehabilitation, or with multidisciplinary rehabilitation alone (for the control group). This approach allows for targeted investigation while maintaining the generalisability of results to a larger population (trial registration: NCT05443100).

### 2.1. Procedure

During the hospitalisation (average length of hospital stay: 26 days), both groups underwent daily 45 min physiotherapy sessions and 45 min supervised standard adapted physical activity sessions. All patients received a balanced, hypocaloric Mediterranean diet consisting of three meals a day with 18–20% protein, 27–30% fat (of which <8% saturated fat) and 50–55% carbohydrates (<15% simple sugars), and 30 g of fibres. In addition to the standard protocol, patients assigned to the RWBC group received a WBC cycle consisting of 10 sessions at −110 °C over a two-week period (Monday to Friday, in the early morning, before physical exercise classes and physiotherapy). During WBC, patients were minimally dressed, wearing a surgical mask, earband, gloves, t-shirt, shorts, socks, plastic clogs, inside the cryochamber (Artic, CryoScience, Rome, Italy). All participants in the RWBC group were given an initial 1 min familiarisation session at −110 °C before starting the research protocol while the following sessions lasted 2 min at −110 °C. Before entering the chamber, any glasses, contact lenses and metal jewellery were removed and the body was dried thoroughly. Vocal and visual contact with the patient was regularly maintained during the session. The patient’s skin surface temperature was measured before and after each treatment with an infrared thermometer (Fluke 62 Max +, Fluke Corporation, Everett, WA, USA) at the neck, quadriceps, popliteal fossa, and calf to confirm the temperature shift after the session.

### 2.2. Measures

#### 2.2.1. Demographic and Clinical Characteristics

Baseline demographic and clinical characteristics were gathered upon admission, encompassing age, gender, body mass index (BMI; calculated as an individual’s weight in kilograms divided by their height in metres squared; kg/m^2^), and NRS score for pain (see Table 1).

#### 2.2.2. Primary Outcome Measures

Twelve months after discharge, a short follow-up telephone interview with dichotomous “YES/NO” response was conducted to investigate the following five domains: well-being status, medication use for pain, fatigue, sleep quality, mood and anxiety. Regarding the domains related to well-being, fatigue, sleep quality, mood and anxiety, the dichotomous questions asked to patients were used to determine whether there had been an improvement after the rehabilitation period (with or without the use of WBC). Regarding medication use after rehabilitation, the dichotomous question helped us to understand whether patients had reduced or discontinued the use of pain-relieving/anti-inflammatory drugs. If the answer was “YES”, an additional question was asked for each dichotomous answer about the number of months the beneficial effects lasted in each area. In addition, the level of pain pre- and post- rehabilitation was also asked and scored with the Numerical Rating Scale (NRS) using a 0 to 10 scale, with zero meaning “no pain” and 10 meaning “the worst pain imaginable” [43]. The questions were designed to adhere to best practices in survey design and clear communication. The full questionnaire is available in the Appendix A.

### 2.3. Statistical Analyses

Prior to the study, we conducted power analyses for the exact Fisher test and the Wilcoxon rank-sum test, choosing a high effect size based on similar studies and clinical experience. Specifically, we aimed for an effect size of 0.35 for the Fisher test and a probability of P (X > Y) of 0.7 for the Wilcoxon test, with a power of 0.8 and an alpha level of 0.05. These calculations indicate that sample sizes of 27 and 33 per group would be required. Although our final sample sizes are somewhat smaller, they are still adequate for detecting statistically significant effects in the sleep and NRS variables, as per our predefined criteria. The effect size for the general well-being variable is closer to the threshold of significance. 

Data collection, visualisation and statistical analyses were performed using R, 4.2.1 version. All continuous data were expressed as mean (median for the time of the positive effects) and standard deviation of the mean. The categorical data were expressed as frequencies and percentages. The Shapiro–Wilk test was carried out to test the normality of the continuous variables.

For the comparison of categorical variables, the Fisher’s test was used. Differences in the time of the positive effects were tested using the Wilcoxon rank sum test with continuity correction (EWSRT). This test was performed to address potential non-normal distributions and to improve the accuracy of our results. The NRS scores were compared between the two groups by using the Mann–Whitney U test. Significance level was set to 0.05 (two-tailed test).

## 3. Results

A higher frequency of patients reported improvements in their general well-being status in the RWBC group (Fisher’s exact test, *p* = 0.022) (22/23, 96%) than in the RG group (15/23, 65%), which lasted 3 months for RWBC and 4 months for RG (all effect duration data in months are expressed as median values). More subjects reduced or discontinued pain medication in the RWBC group (13/22 patients, 59%, for a median of 5 months), but this did not differ significantly from the RG group (8/21 patients, 42%, for a median of 5 months; Fisher’s exact test, *p* = 0.2271). The majority of patients in both groups (21/23, 91% in the RWBC group and 18/23, 78% in the RG group) noted increased energy and decreased general fatigue up to 3 months for both the RWBC and RG group after treatment discontinuation, but the difference between the two groups was not statistically significant (*p* = 0.414). 

More RWBC subjects reported better sleep quality (14/22, 64%), with positive effects lasting for a median of 3.5 months, compared to RG subjects (*p* = 0.001), among which only 2 out of 19 (11%) patients reported improvements that lasted, respectively, 1 and 3 months.

**Figure 2:**
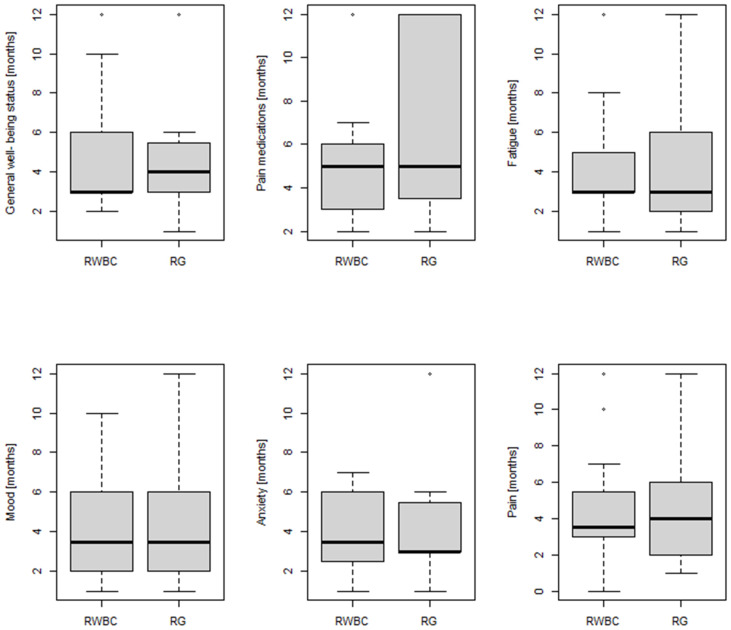
Comparison between the two groups (RWBC vs. RG) on the duration (in months) of positive effects on general well-being, pain medications, fatigue, sleep quality, mood and anxiety.

Patients who showed improvement in mood (14/16, 88% in RWBC vs. 16/18, 89% in RG) and anxiety (12/16, 75% in RWBC vs. 12/18, 67% in RG) were similar in both groups (*p* = 1.000 and *p* = 0.715, respectively). Regarding mood, the beneficial effects lasted for a median of 3.5 months in both groups, and as for anxiety, the effects lasted for a median of 3.5 months in the RWBC group and 3 months in the RG group. All results are shown in Table 2 and Figure 2. 

**Figure 3:**
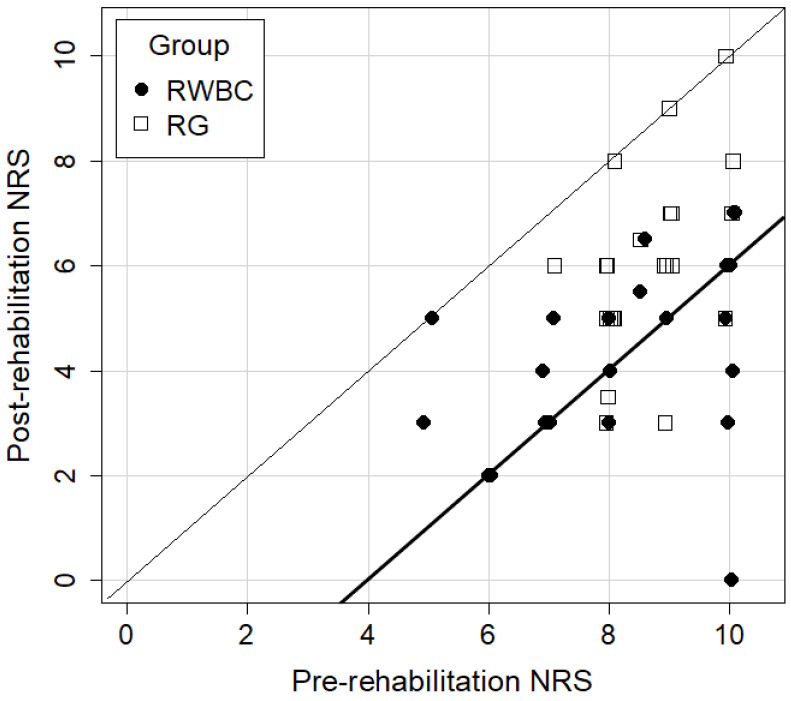
A scatter plot (jittered) of the improvement in pre- and post-rehabilitation NRS scores in the two groups (RWBC vs. RG). Individuals located between the thin and thick diagonal lines experienced a decrease in pain ranging from 0 to 4 points. Those located below the thick line experienced a decrease in pain of more than 4 points. The distance of the point from the thin diagonal is therefore proportional to the change in NRS scores.

**Figure 4:**
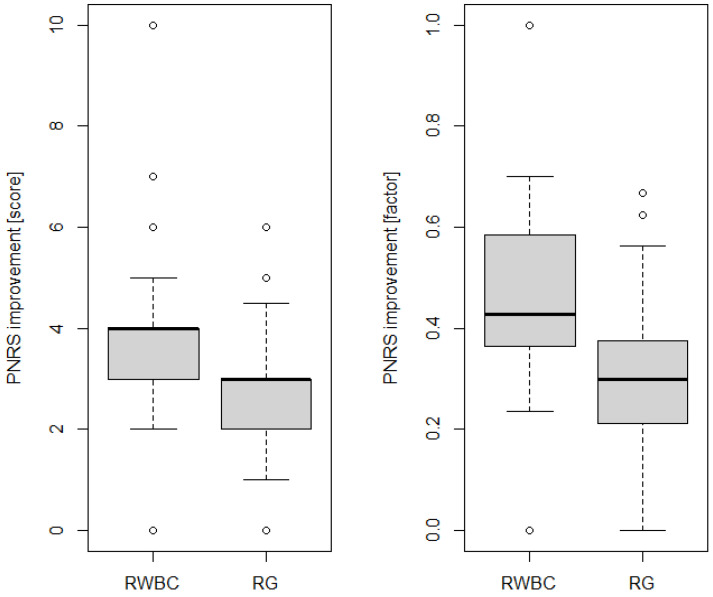
The boxplots for comparing the improvement of two groups (RWBC vs. RG), shown as the absolute difference between the pre- and post-rehabilitation NRS scores (**left**), and as a factor (difference divided by the pre-rehabilitation score; **right**).

Finally, both groups showed an improvement in pain scores post-rehabilitation compared to the pre-rehabilitation scores (*p* < 0.001), but the improvements were significantly higher in the RWBC group compared to the RG (Mann–Whitney U test, *p* = 0.002, see Figure 3 and Figure 4), with effects lasting, respectively, for a median of 3.5 and 4 months (see Figure 2). 

## 4. Discussion

The use of WBC in alleviating pain and improving health-related quality of life in FM patients has been previously investigated in the literature [36,37,38]. A recent study by our research group [9] analysed the effects of WBC in patients with FM and obesity, showing that the addition of 10 WBC sessions within a multidisciplinary rehabilitation program resulted in greater benefits in pain severity, depressive symptomatology, disease impact, and sleep quality at discharge as compared to the control group undergoing only the rehabilitation program. Only two studies have investigated the duration of the effects of 10 WBC sessions in those patients. Vitenet et al. [39] observed on 24 FM patients that the positive effects of 10 WBC sessions and rehabilitation provided over 8 days, as assessed by the physical and mental composite scores of SF-36, lasted for at least one month following the interventions. Klemm et al. [35] showed that 3 months after WBC discontinuation, the effects on pain and disease activity were no longer present. Specifically, they demonstrated that serial WBC (between 6 and 10 sessions in a maximum of 3 weeks) elicited effects lasting more than 1 month after ceasing WBC treatment, then decreasing gradually to null effect after 3 months. These patients, however, were not followed up thereafter.

To obtain information on the duration of clinical–functional benefits of WBC after 10 treatments, we decided to conduct a simple follow-up telephone interview 12 months after discharge on FM patients referred to our hospital for a multidisciplinary rehabilitation program and to compare them with those obtained in patients who followed the same multidisciplinary rehabilitation program but without WBC. We structured our telephone interview in order to obtain first dichotomous responses (YES/NO) and then the number of months, the beneficial effects lasted and the NRS score to assess level of pain. 

More patients in the RWBC group reported improvements in their general well-being and sleep quality compared to the RG group, with an average duration of the effects lasting about 3–4 months. Moreover, the improvements in NRS scores for pain were significantly higher in the RWBC group compared to the RG. However, the feedback provided for general fatigue, mood and anxiety was not different between the two groups. In fact, this study shows that both groups reported positive effects on many outcomes after rehabilitation, but the implementation of WBC within the multidisciplinary program was more effective in managing some of the major symptoms of FM, such as pain and sleep quality. Positive effects of a WBC cycle on pain level, sleep quality and depressive symptoms have also been observed in other studies [28,37,40], although with different study designs. Thus, our results are in line with the study by Varallo et al. [9], where the group undergoing WBC and rehabilitation showed greater improvements in the severity of pain, depressive symptoms, and quality of sleep compared to the group undergoing rehabilitation alone. We also saw greater improvements in pain level and sleep quality in the RWBC group than in the control group, while no significant differences were found between the two groups with regard to mood and anxiety parameters. This discrepancy could be partly explained by the fact that we conducted our follow-up telephone interview one year after discharge, which may have challenged patients’ memory and reliability of responses. Also, due to the telephone nature of the interview, we opted for a short format with simple questions, which may not have the same accuracy as a validated questionnaire.

Some limitations must be taken into consideration. First, the multidisciplinary rehabilitation program included nutritional interventions, physiotherapy, physical activity, and psychological support, making it difficult to estimate to which extent WBC per se contributed to symptom management. Secondly, as mentioned before, telephone interviews were conducted one year after the patients were discharged, which poses challenges to the accuracy of the answers provided. 

Another notable limitation of our study are the sample sizes, which, due to unforeseen challenges in data collection, were slightly smaller than initially planned. However, our final sample sizes were sufficient for the variables deemed statistically significant under our study conditions.

Furthermore, having more than one primary outcome measure changes the *p*-value needed for significance. A more rigid significance threshold, such as a Bonferroni-corrected *p*-value, could be considered. Despite the application of such a correction, our results would remain virtually unchanged, with the only difference observed in the general well-being variable. Nonetheless, given that the only preliminary measurement (pre) included was the NRS pain score, and that the NRS pain score after treatment (post) showed significant amelioration (improvement *p* < 0.001; comparison *p* = 0.002) compared with the control group, this supports the notion that the overall results are clinically meaningful. 

Future research will aim to implement follow-up interviews to evaluate the mid- and long-term effects of different WBC protocols in larger FM populations to provide further insights and improve the robustness of our results. In addition, WBC studies will be needed to better adjust the number of sessions, exposure duration, and chamber temperature to optimize clinical–functional outcomes.

## 5. Conclusions

Managing FM symptoms is a frustrating condition for patients, and the fact that its aetiology is unknown makes it difficult for physicians to diagnose. Our findings that treatment with WBC is effective and provides lasting improvements are an incentive to develop and study similar treatment programs.

Our preliminary data show that a nonpharmacological strategy such as WBC can increase the positive effects of rehabilitation interventions on FM patients’ general well-being, pain level, and sleep quality, with an average duration of effects of about 3–4 months. Therefore, in the future, larger follow-up studies will need to unravel the frequency of repeated cycles of WBC to maximise the long-term effects in the management of FM symptoms. Cost-effectiveness studies will also be needed to consider the implementation of WBC in FM rehabilitation protocols. 

## Figures and Tables

**Table 1:** Characteristics of the population at baseline, divided into the two study groups.

	*n*	Age (Mean ± SD)	BMI (Mean ± SD)	NRS Pre-Rehabilitation Score (Mean ± SD)
RWBC	23	57.5 ± 8.9	41.1 ± 7	8.3 ± 1.7
RG	23	57 ± 8	40 ± 5.7	8.6 ± 0.8

NOTE. *n*—number of the included patients; BMI—Body Mass Index; and NRS—Numerical Rating Scale.

**Table 2:** Percentage (%) of patients by group (RWBC vs. RG) who answered YES for each item considered.

		RWBC	RG	*p* Value **
Well-being status	*n*	23	23	0.022 *
	*n*_yes_ (%)	22 (96%)	15 (65%)	
Pain medications	*n*	22	21	0.227
	*n*_yes_ (%)	13 (59%)	8 (38%)	
Fatigue	*n*	23	23	0.414
	*n*_yes_ (%)	21 (91%)	18 (78%)	
Sleep Quality	*n*	22	19	0.001 *
	*n*_yes_ (%)	14 (64%)	2 (11%)	
Mood	*n*	16	18	1.000
	*n*_yes_ (%)	14 (88%)	16 (89%)	
Anxiety	*n*	16	18	0.715
	*n*_yes_ (%)	12 (75%)	12 (67%)	

NOTE. Values are *n* (%) for YES; ** Fisher’s exact test; * indicates *p* values that are statistically significant.

## Data Availability

Data are available on request in Zenodo repository (https://doi.org/10.5281/zenodo.11565945) due to restrictions, e.g., privacy or ethical.

## Appendix A

Below are the questions asked to patients during the telephone interview (original Italian version/*English translation*):

Stato di benessere/*Well-being status*: Ha notato qualche beneficio in termini di benessere generale dopo aver completato i trattamenti di WBC/il periodo di riabilitazione? (Se la risposta è SI) Per quanto tempo (in mesi) sono durati questi benefici?/*Did you notice any benefits in terms of general well-being status after completing the WBC treatments/the rehabilitation period? (If the answer is YES) How long (in months) did these benefits last?*

Utilizzo di farmaci per il dolore/*Medication use for pain*: Dopo aver terminato i trattamenti di WBC/la riabilitazione, ha interrotto o ridotto l’assunzione di farmaci antidolorifici/antinfiammatori? (Se la risposta è SI) Per quanto tempo (numero di mesi)?/*After finishing WBC treatments/rehabilitation, did you stop or reduce your intake of pain-relieving/anti-inflammatory medications? (If the answer is YES) For how long (number of months)?*

Dolore/*Pain*: Prima di iniziare la WBC/la riabilitazione, quale era la sua scala del dolore da 0 a 10, dove 0 rappresenta l’assenza di dolore, mentre 10 rappresenta il massimo dolore? Dopo aver concluso i trattamenti di WBC/la riabilitazione, quale era la sua scala del dolore da 0 a 10, dove 0 rappresenta l’assenza di dolore, mentre 10 rappresenta il massimo dolore? (Se la scala del dolore è migliorata) Per quanto tempo (numero di mesi) ha avuto benefici sul dolore?/*Before you started WBC/rehabilitation, what was your pain scale from 0 to 10, where 0 represents no pain and 10 represents maximum pain? After completing the WBC treatments/rehabilitation, what was your pain scale from 0 to 10, where 0 represents no pain and 10 represents maximum pain? (If the pain score has improved) For how long (in months) have you experienced pain relief?*

Fatica/*Fatigue*: Dopo i trattamenti di WBC/la riabilitazione, ha notato dei benefici sulla fatica? (Se la risposta è SI) Per quanto tempo (numero di mesi)? *After WBC treatments/rehabilitation, have you noticed any benefits on fatigue? (If answer is YES) For how long (number of months)?*

Qualità del sonno/*Sleep quality*: Dopo i trattamenti di WBC/la riabilitazione ha avuto dei benefici sulla qualità del sonno? (Se la risposta è SI) Per quanto tempo (numero di mesi)?/*After the WBC treatments/rehabilitation, have you experienced any benefits on sleep quality? (If the answer is YES) For how long (number of months)?*

Umore/*Mood*: Dopo aver concluso la WBC/la riabilitazione, ha avuto dei benefici sull’umore? (Se la risposta è SI) Per quanto tempo (numero di mesi)?/*After completing WBC/rehabilitation, did you experience any benefits on your mood? (If the answer is YES) For how long (number of months)?*

Ansia/*Anxiety*: Dopo aver concluso la WBC/la riabilitazione, ha avuto dei benefici sull’ansia? (Se la risposta è SI) Per quanto tempo (numero di mesi)?/*After completing WBC/rehabilitation, did you experience any benefits on anxiety? (If the answer is YES) For how long (number of months)?*
